# Microchannels Formed Using Metal Microdroplets

**DOI:** 10.3390/mi14101922

**Published:** 2023-10-10

**Authors:** Daicong Zhang, Chunhui Jing, Wei Guo, Yuan Xiao, Jun Luo, Lehua Qi

**Affiliations:** 1 School of Mechanical & Electronic Engineering, Xi’an Polytechnic University, Xi’an 710048, China; 18220591109@163.com (C.J.); gw13429775696@163.com (W.G.); xiaoyuanjidian@xpu.edu.cn (Y.X.); 2 School of Mechanical Engineering, Northwestern Polytechnical University, Xi’an 710072, China; luojunshaan@gmail.com

**Keywords:** microstructure production, VOF method, microchannel parts metal, microdroplet deposition

## Abstract

The metal microdroplet deposition manufacturing technique has gained extensive attention due to its potential applications in microstructure fabrication. In order to fabricate components such as microchannel heat sinks and microchannel reactors, this paper investigates the interactions and influences between microdroplets and substrates, as well as between microdroplets themselves. The transient phenomena during the fusion of metal microdroplets in contact with the substrate and the formation of inclined columns, as well as the solid–liquid coupling and morphology formation processes during the collision between microdroplets, are analyzed. The influence of microdroplet spacing on the morphology of microchannels during their formation is specifically studied. A three-dimensional finite element numerical model for the deposition of metal microdroplets forming inclined pillars is established based on the volume of fluid (VOF) method. The model treats the protective gas around the microdroplet as an empty zone and the microdroplet as a single-phase fluid. Simulation analysis is conducted to investigate the forming patterns of unsupported microdroplets at different spacing and their impact on the fusion morphology of microchannel components. Building upon this, a series of validation experiments are conducted using a piezoelectric microdroplet generator to produce uniform aluminum alloy microdroplets with a diameter of approximately 600 μm. A method for fabricating metal microchannel structures is obtained, which is expected to be applied in fields such as scattering structures for high-power electronic devices and microreactors in microchemical fields.

## 1. Introduction

Metal microdroplet deposition is used as a process method in additive manufacturing technology, a technology that allows the direct use of common metallic materials as raw materials [1]. The use of molten microdroplets at high and low frequencies can achieve the precise deposition of aluminum and tin-lead materials, and scanning deposition can be used for stacking larger objects or materials [2]. The freedom of the shapes of metal parts that can be manufactured is greatly extended [3,4]. In addition, the technique has many potential applications in metals with porosity and microfluidic channels or in lattice-structured heat sinks with enhanced thermal properties [5,6]. The use of microchannel heat sinks has also been proposed in recent years [7]. However, several geometric control issues persist, such as parameter overlap between adjacent microdroplets, contour height, and contour closure. These issues can impact the sealing and accuracy of the formed parts. In the case of microchannel components fabricated using metal melt microdroplet deposition, the selection of microdroplet spacing plays a crucial role in determining the morphological features of the microchannels and provides valuable guidance. Therefore, to fabricate microchannel components that meet the required physical properties, altering the microdroplet deposition spacing and leveraging the resulting oscillation phenomena can be employed to control the shape of the microchannel components.

The principle of solder microdroplet printing technology and its application in the microelectronics industry was first proposed in 2001 [8]. In recent years, several rapid prototyping systems based on metal microdroplets have been developed to produce various parts. Metal melt microdroplets can be directly deposited to create straight, circular, triangular, and other patterns. Further deposition can be conducted to establish vertical pillars [9,10]. As the research on microchannel performance and structure progresses, the complexity of microchannel shaping requirements continues to increase. The core of optimizing microchannels lies in analyzing heat conduction during the shaping process and understanding the effects of microdroplet–substrate fusion and microdroplet–microdroplet fusion on the shaping state [11,12,13]. R. Pastuszko et al. from the California Institute of Technology compared the pool boiling heat transfer of microfin-enhanced structures with porous and non-porous covers to identify optimal parameters for perforated foils or wire-mesh-sintered microfins with the highest heat transfer coefficient [14]. Yoav Peles et al. from Rensselaer Polytechnic Institute investigated flow boiling in micro-pin-fin and circular staggered micro-pin-fin heat sinks [15,16]. W. Qu et al. from the University of Hawaii studied water-saturated flow boiling heat transfer in a staggered square micro-pin array [17]. H. Pehlivan et al. from Sakarya University in Turkey conducted experimental research on heat transfer rates in sinusoidal-wave-shaped channels, comparing three different types of peaked-wave fin structures with a plain surface microchannel [18]. M.A. Ahmed et al. from the University of Malaya performed numerical studies on the laminar forced convective heat transfer of copper–water nanofluid in trapezoidal corrugated channels, analyzing the effects of wave amplitude, wavelength, nanoparticle volume fraction, and Reynolds number on velocity vectors, temperature contours, pressure drop, and average Nusselt number [19]. Z. Wan et al. from South China University of Technology investigated the two-phase heat transfer characteristics of semi-corrugated microchannels, observing the transition of bubbly flow to slug flow, churn flow, and annular flow as the effective heat flux density increased. Semi-corrugated microchannels exhibited higher heat transfer coefficients and lower superheat temperatures during boiling processes compared to flat microchannels under effective heat flux density conditions [20].

Microchannel parts are usually machined and fabricated using high-speed milling processes, microcasting, post-stamping welding, and etching. Ewa Raja et al. of Lodz Institute of Technology, USA, used a wire-cutting process and a milling process to obtain different microchannel shapes to further analyze the effect of microchannel shape, geometrical parameters, and thermal parameters on the cooling performance of microchannel structures [21]. Muammer et al. of Virginia Commonwealth University, USA, used hybrid microfabrication to prepare large-area multi-array microchannel fuel cell bipolar plates using embossing and welding processes [22]. M. Law et al. of the National University of Singapore prepared copper slant fin microchannel heat sinks by introducing slant fins modified from conventional straight fins using an EDM wire-cutting process and performed boiling experiments [23]. Wei Wan et al. of Xiamen University used a laser micro-milling method to fabricate miniature tapered pin tabs on the bottom surface of rectangular microchannels and studied the structured microchannels with micro-pin fins for advanced microchannel heat sinks to cool high-heat-flow devices. The experiments compared the flow boiling performance of the micro-pin-fin-structured microchannels with the conventional rectangular microchannels with smooth bottom wall surface [24]. Deng Daxiang et al. of South China University of Technology prepared a new porous microchannel heat sink using a conventional sintering process and investigated the two-phase boiling heat transfer performance of re-enterable porous microchannels to explore the feasibility of the application of strengthening techniques in heat sink cooling [25]. Zhang Canbin et al. of Shenzhen University used a micro-stamping process to prepare metal bipolar plates, and introduced a flexible forming process–polymer powder media flexible forming method [26]. The conventional process can only prepare microchannels with a simple inner wall structure, and most of them are spliced in a complex process with little customization and flexibility.

The use of metal microdroplet injection molding technology provides a new way to manufacture tiny thin-walled complex aluminum alloy parts. Currently, microdroplet collisions can be simulated using a free surface method, which is generally classified as a dynamic mesh method and a static mesh method. In the dynamic mesh method, the boundary of the mesh is the free surface of the fluid. The computational nodes move with the fluid. Finite element computational techniques are usually applied to the solution of the dynamic mesh. Waldvogel and Poulikakos developed a two-dimensional finite element model to study microdroplet collisions and solidification. Different material properties of the liquid and solid phases were used in his model [27]. Real-time surface tension coefficients and velocities were applied to the finite element model by Rajneesh Bhardwaj et al., who investigated Marangoni convection and variable speed phenomena in the solder microdroplet deposition process [28]. Subsequently, Carlson et al. proposed a dynamic wetting modeling approach based on phase field theory to determine a new wetting state during the microdroplet diffusion phase [29], and this model can describe the wetting effect between the microdroplet and the substrate. However, when the fluid undergoes greater deformation, the mesh must be re-divided. In the static mesh approach, the mesh cannot deform with the fluid. Surface tracking techniques, such as VOF and level set methods, require the calculation of the coordinates of the free surface [30] and the VOF method is one of the methods widely used in modeling free surfaces. Li et al. used the VOF method to study the spreading and rebound process of a liquid microdroplet on a planar solid [31]. In addition to microdroplet collisions on horizontal surfaces, microdroplet collisions on inclined surfaces have also been encountered in engineering applications. Pasandideh-Fard et al. developed a three-dimensional microdroplet collision and solidification model on an inclined substrate capable of matching well with experiments [32]. Qi et al. investigated the trajectory planning for aluminum microdroplet deposition when deposited on different cross sections [33]. The substrate surfaces are flat in the above discussion, both for normal and inclined substrates. However, in practice, the substrate surface where microdroplets collide may not be perfectly flat or regular in shape. Experiments have shown that even small irregularities on the surface can have a large dynamic collision effect. Unfortunately, very little information has been reported in the literature on microdroplet collisions on non-flat substrates.

Although microdroplet collisions on regular or even irregular solid surfaces have been studied more frequently, the current studies are still deficient in terms of microdroplet-to-microdroplet collision and remelting mechanisms due to the high surface tension, fusion temperature, latent heat of solidification, and high thermal conductivity of molten metals, which involve more complex liquid–solid coupling and remelting solidification. There are some reported studies on the deposition of vertical columns by microdroplets on horizontal substrates. For example, Du et al. analyzed the transient transport phenomenon in the deposition of metal microdroplets on substrates [34]. Qi et al. filled an experimental system with a nitrogen source and used a solenoid controller nozzle to form aluminum alloy microdroplets to fabricate parts. The authors predicted and experimented with the remelting and bonding of aluminum alloy three-dimensional parts under different process parameters, and investigated the effect of different microdroplet temperatures and substrate temperatures on sample formation [35]. Zhang et al. developed an experimental system for the direct deposition of aluminum microdroplets under unsupported conditions and proposed a heat transfer model that can be used to distinguish the degree of microdroplet melting at different offsets [13].

However, no simulation study has been conducted of tilted column formation using metal microdroplet deposition, and in microdroplet additive manufacturing technology, the ability to form tilted columns at a certain angle enables the formation of more complex geometries. Finite element methods are widely used to study the specific effects of various processing parameters on the forming quality of the final machined part. In this article, the process of continuous aluminum microdroplet deposition is modeled and analyzed using the VOF method with the help of Flow-3D software (v11.2). A three-dimensional numerical model of a metal microdroplet deposited on an inclined column is developed to analyze the liquid–solid coupling, heat transfer, and deposition morphology during the microdroplet collision process. The flow and thermal behavior of the microdroplet collision process is analyzed and discussed under different parameters. Finally, aluminum microdroplet channel parts formed at different pitches are prepared, and the microchannel part structures are observed to demonstrate the potential feasibility of preparing metal-microdroplet-deposited microchannel parts.

## 2. Modeling and Experimental Setup

### 2.1. Control Equations

The calculation of the volume change and temperature field of the microdroplet deposition process is a microdroplet typical nonlinear transient heat transfer solution problem. The state of the control volume is marked by the fluid volume function defining a percentage F of the liquid phase. The control volume is all F *=* 1 when the control volume is entirely in liquid phase, F *=* 0 when the control volume is entirely in solid phase, and F is between 0 and 1 when the control volume is partially in liquid phase. F satisfies the volume function equation:

(1) $$\frac{\partial F}{\partial t}+\frac{1}{V_{F}}\left[\frac{\partial }{\partial x}\left({\mathrm{FA}}_{x}u\right)+\frac{\partial }{\partial y}\left({\mathrm{FA}}_{y}v\right)+\frac{\partial }{\partial z}\left({\mathrm{FA}}_{z}w\right)\right]=0$$

u, v, and w are the velocity components in the x, y, and z directions in the Cartesian coordinate system, respectively. A_x_, A_y_, and A_z_ are the fluid area fractions in the x, y, and z directions, respectively.

The mass continuity equation is:

(2) $$V_{F}\frac{\partial \rho }{\partial t}+\frac{\partial }{\partial x}\left({\rho \mathrm{uA}}_{x}\right)+\frac{\partial }{\partial y}\left({\rho \mathrm{vA}}_{y}\right)+\frac{\partial }{\partial z}\left({\rho \mathrm{wA}}_{z}\right)=0$$

V_F_ is the volume fraction occupied by the fluid in the cell and ρ is the density of the metal microdroplet. The momentum conservation equations for the fluid velocity components (u, v, w) are:

(3) $$\frac{\partial u}{\partial t}+\frac{1}{V_{F}}\left({\mathrm{uA}}_{x}\frac{\partial u}{\partial x}{+\mathrm{vA}}_{y}\frac{\partial v}{\partial y}{+\mathrm{uA}}_{z}\frac{\partial w}{\partial z}\right)=\frac{1}{\rho }\frac{\partial p}{\partial x}{+G}_{x}{+S}_{x}-{\mathrm{Ku}}_{x}$$

(4) $$\frac{\partial v}{\partial t}+\frac{1}{V_{F}}\left({\mathrm{uA}}_{x}\frac{\partial u}{\partial x}{+\mathrm{vA}}_{y}\frac{\partial v}{\partial y}{+\mathrm{uA}}_{z}\frac{\partial w}{\partial z}\right)=\frac{1}{\rho }\frac{\partial p}{\partial y}{+G}_{y}{+S}_{y}-{\mathrm{Ku}}_{y}$$

(5) $$\frac{\partial w}{\partial t}+\frac{1}{V_{F}}\left({\mathrm{uA}}_{x}\frac{\partial u}{\partial x}{+\mathrm{vA}}_{y}\frac{\partial v}{\partial y}{+\mathrm{uA}}_{z}\frac{\partial w}{\partial z}\right)=\frac{1}{\rho }\frac{\partial p}{\partial z}{+G}_{z}{+S}_{z}-{\mathrm{Ku}}_{z}$$

(G_x_, G_y_, G_z_) is the volume force acceleration, (S_x_, S_y_, S_z_) is the surface tension, and (Ku_x_, Ku_y_, Ku_z_) is the coagulation resistance, where K is the resistance coefficient. The Darcy-type solidification resistance model is used here, and the expression for K is as follows:

(6) $$K=\frac{F_{S}^{2}}{{\left(1-F_{S}\right)}^{3}}$$

F_s_ is the solid phase fraction and its expression is:

(7) $$F_{S}=\left\{\begin{array}{c} 0\\ \frac{T_{\mathrm{liquidus}}-T}{T_{\mathrm{liquidus}}-T_{\mathrm{solidus}}}\;\\ 1 \end{array}\right.\begin{array}{cc} \mathrm{if} & {T\;>\;T}_{\mathrm{liquidus}}\\ \mathrm{if} & T_{\mathrm{solidus}}{\;<\;T\;<\;T}_{\mathrm{liquidus}}\\ \mathrm{if} & {T\;<\;T}_{\mathrm{solidus}} \end{array}$$

where T_liquidus_ and T_solidus_ are the solid–liquid phase line temperatures of the metal microdroplets, respectively.

(S_x_, S_y_, S_z_) satisfies the following equation:

(8) $$S_{x}=\sigma \int {\cos\theta }_{x}\left[\frac{\mathrm{dx}}{{\cos\Theta }_{x}}\right]$$

(9) $$S_{y}=\sigma \int {\cos\theta }_{y}\left[\frac{\mathrm{dy}}{{\cos\Theta }_{y}}\right]$$

(10) $$S_{z}=\sigma \int {\cos\theta }_{z}\left[\frac{\mathrm{dz}}{{\cos\Theta }_{z}}\right]$$

where σ is the surface tension coefficient, (θ_x_, θ_y_, θ_z_) is the angle between the second principal tangent and the x, y, and z axes, respectively, and (Θ_x_, Θ_y_, Θ_z_) is the angle between the first principal tangent and the x, y, and z axes, respectively.

The energy conservation equation is:

(11) $$V_{F}\frac{\partial }{\partial t}\left(\rho I\right)+\frac{\partial }{\partial x}\left({\rho \mathrm{IuA}}_{x}\right)+\frac{\partial }{\partial y}\left({\rho \mathrm{IvA}}_{y}\right)+\frac{\partial }{\partial z}\left({\rho \mathrm{IwA}}_{z}\right)\;=-p\left(\frac{\partial {\mathrm{uA}}_{x}}{\partial x}+\frac{\partial {\mathrm{vA}}_{y}}{\partial y}+\frac{\partial {\mathrm{wA}}_{z}}{\partial z}\right){+T}_{\mathrm{DIF}}$$

where I is the macroscopic internal energy, expressed as a function of temperature:

(12) $${I=C}_{l}\cdot T+\left(1\;-{\;F}_{s}\right)\cdot C_{\mathrm{LHT}}$$

where C_l_ is the specific heat capacity of the metal microdroplet and C_LHT_ is the latent heat of solidification.

The thermal diffusivity T_DIF_ is:

(13) $$T_{\mathrm{DIF}}=\frac{\partial }{\partial x}\left({\mathrm{kA}}_{x}\frac{\partial T}{\partial x}\right)+\frac{\partial }{\partial x}\left({\mathrm{kA}}_{y}\frac{\partial T}{\partial y}\right)+\frac{\partial }{\partial z}\left({\mathrm{kA}}_{z}\frac{\partial T}{\partial z}\right)$$

where k is the thermal conductivity of the metal microdroplet.

The heat transfer between the metal microdroplet and the substrate is:

(14) $$\begin{array}{l} \left(1-V_{F}\right)\rho_{sub}C_{sub}\frac{\partial T_{sub}}{\partial t}-\frac{\partial }{\partial x}\left[k_{sub}\left(1-A_{x}\right)\frac{\partial T_{sub}}{\partial x}\right]-\\ \frac{\partial }{\partial y}\left[k_{sub}\left(1-A_{y}\right)\frac{\partial T_{sub}}{\partial y}\right]-\frac{\partial }{\partial z}\left[k_{sub}\left(1-A_{z}\right)\frac{\partial T_{sub}}{\partial z}\right]=T_{SOR} \end{array}$$

T_sub_ is the substrate temperature, and ρ_sub_, C_sub_, and k_sub_ are the density, specific heat capacity, and thermal conductivity of the substrate, respectively. T_SOR_ is the heat transfer between the fluid and the substrate. The heat passing through the unit interface within the grid cell can be expressed as:

(15) $$q=hS_{A}\left(T_{sub}-T\right)$$

h is the heat transfer coefficient between the microdroplet and the substrate, S_A_ is the interface area within the grid cell, and T and T_sub_ are the temperatures of the metal microdroplet and the substrate surface, respectively.

### 2.2. Volume of Fluid Method Modeling

The microdroplet continuous deposition model consists of two parts: the microdroplet and the substrate. The microdroplet diameter is 0.6 mm and the vertical drop velocity is 0.35 m/s. The protective gas around the microdroplet is considered an empty domain, thus turning the model into a single-phase flow. Due to the small microdroplet size and low drop velocity, the influence of the gas around the microdroplet is small, and treating it as an empty domain has little effect on the calculation results. Related studies have shown that the heat dissipated by radiation from the microdroplet through the upper surface of the microdroplet is less than 10% of the heat transferred from the microdroplet to the substrate, so the radiation heat dissipation from the upper surface of the microdroplet is neglected in this model [36,37,38]. Pure aluminum is used in the simulations and its thermal properties are listed in Table 1. Theoretically, the solidification of pure aluminum occurs at its melting point, but the solid–liquid phase line temperature range is set to 1 K in this model for the accuracy of the calculations.

The microdroplet parameters can be controlled by a defined subroutine in which all parameters of the microdroplet, such as number, generation position, size, temperature, speed, and time, can be set. In this model, the initial velocity of the microdroplets is along the vertical direction and the initial temperature of the microdroplets is 960 K. The molding is controlled by adjusting the distance between the substrate and the injection device. The contact thermal resistance between the microdroplet and the substrate varies with time and position, but is set to 1 × 10^−6^ m^2^·K/W for the convenience of calculation, corresponding to a heat transfer coefficient of 1 × 10^6^ W/(m^2^·K), i.e., the microdroplet is in ideal contact with the substrate. The boundary condition of the computational domain is set to continuous boundary, which means that the fluid passes smoothly and continuously through the boundary. Let the contact angle between the microdroplet surface and the solid wall be 90°.

**Figure 1:**
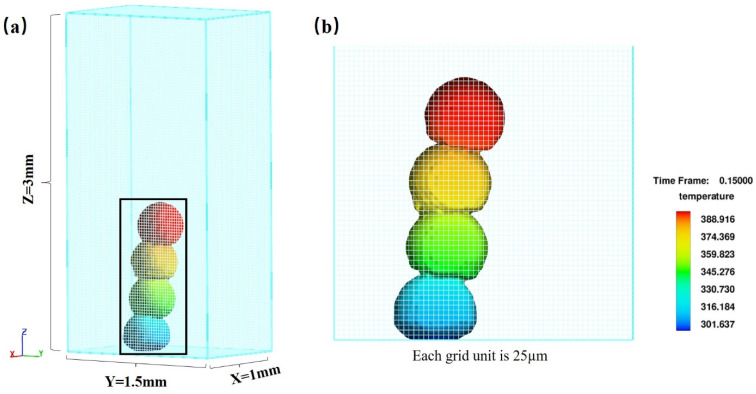
(**a**) Schematic diagram of mesh division within Flow-3D. (**b**) Schematic diagram of the number of grids occupied by microdroplets during simulation.

The FAVOR (Fractional Area/Volume Obstacle Representation) technique in Flow-3D was used for the mesh dissection. The protective gas around the microdroplet is considered an empty area, thus turning the model into a single-phase flow. Due to the small size of the microdroplet and the low fall velocity, the gas around the microdroplet is affected very little and treating it as an empty domain has little effect on the calculation results. It has been shown that the heat dissipated by radiation from the upper surface of the microdroplet is less than 10% of the heat transferred from the microdroplet to the substrate, so the radiation heat dissipation from the upper surface of the microdroplet is neglected in this model. In order to capture the rapid change in the microdroplet morphology, there are 40 meshes for the X-axis direction of 1 mm, 60 meshes for the Y-axis direction of 1.5 mm, and 120 meshes for the Z-axis direction of 3 mm, each with a length of 0.025 mm, totaling 288,000 meshes, as shown in Figure 1. The total operation time is 0.15 s, and the operation step length is 0.0001 s per step, which is 1500 steps in total.

The effect of the wettability of the substrate on deposition is mainly seen in the size of the contact angle after molding, which varies from substrate to substrate, presenting a different contact angle. The final contact angle presented is also affected by other factors such as droplet temperature, ambient temperature, substrate temperature, and surface oxidation. Usually, the size of the contact angle is measured experimentally as a process parameter under specific factors. In this simulation experiment, the substrate chosen is a smooth aluminum room-temperature metal plate.

## 3. Simulation Analysis

### 3.1. Microchannel Molding Process

**Figure 2:**
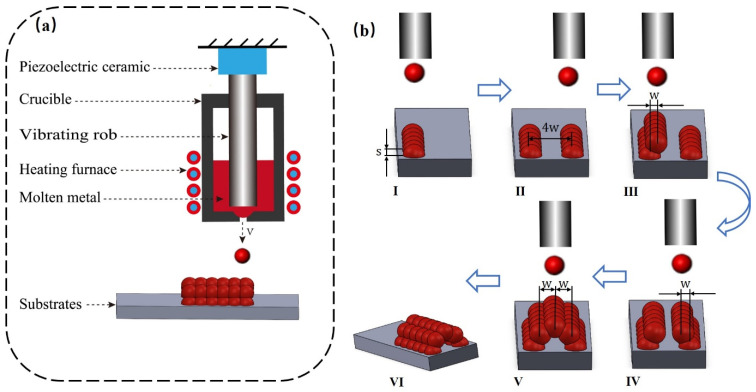
(**a**) Schematic diagram of microchannel deposition using a metal-based microdroplet injection platform. (**b**) Process principle of depositing microchannels on a substrate (I: depositing the left row of microchannels (s is the fixed spacing when depositing each row of microchannels); II: depositing the second row far from the first row 4w (w is the interval between microdroplets in each layer); III: returning to the first row and moving w far to the right to deposit the third row; IV: returning to the second row and moving w far to the left to deposit the fourth row; V: continuing to move w far to the left to deposit the fifth row; VI: microchannel molding is complete).

Figure 2a shows a schematic diagram of a metal microdroplet injection molding microchannel. It consists of a DOD generator based on a piezoelectric ceramic and a vibrating cavity, a heating system, and a microdroplet storage deposition system. The microdroplets are deposited onto the substrate after heating by the heater, and the microdroplets are gradually reduced to the substrate temperature. After the microdroplets are cured, the next microdroplet is deposited at a predesigned location, and the procedure is repeated to complete the microchannel formation. Figure 2b shows the whole process of depositing microchannels. The microchannel-forming stage is divided into five steps, and the distance of each row of microdroplets is controlled for deposition.

### 3.2. Analysis of the Dynamic Process of Aluminum Microdroplet Deposition

Depending on the morphology and thermal conductivity of the bottom surface of the microdroplet deposition, there are two cases: in the first case, the microdroplet is deposited directly onto a flat substrate; in the second case, the microdroplet is deposited onto a microdroplet that has already solidified or is about to solidify. In the first case, the surface on which the microdroplets are deposited is generally flat and the substrate can be considered an infinite thermal conductor; in the second case, the surface on which the microdroplets are deposited is a spherical crown formed by the microdroplets, and the heat is dissipated by the substrate through the microdroplets in contact with the substrate and the heat transfer model can be regarded as a one-dimensional thermal conductivity problem with a fused interface for the heat transfer cross section.

The main forces acting in the microdroplet deposition process include inertial forces, gravity, viscous forces, and surface tension. Among them, the inertial force and surface tension play an important role in the evolution of the microdroplet morphology during the microdroplet deposition process. The ratio of inertial force to surface tension is defined as the Weber number and the ratio of viscous force to surface tension is defined as the Ohnesorge number, whose expression (16) is:

(16) $$\mathrm{Oh}=\frac{\mu_{1}}{\sqrt{\sigma_{1}\rho_{1}D_{j}}}$$

where μ_1_—fluid viscosity, ρ_1_—fluid density, σ_1_—surface tension coefficient, and d_1_—microdroplet diameter.

According to the initial conditions of the microdroplet, We = 1.7, Oh ≪ 1, so the microdroplet belongs to the collision-dominated mode without viscosity; that is, the initial spreading of the microdroplet is rapid, mainly dominated by the inertial force and surface tension, and the main resistance is the inertia of the microdroplet. As the spreading speed decreases, the role of viscous force is more significant.

In this simulation, only the offset of the microdroplet when falling was changed, and the variation in the aluminum alloy with a microdroplet diameter of 600 μm deposited at 960 K with a falling speed of 0.35 m/s on a substrate at 300 K was simulated.

The relevant parameters for the experimental simulations are shown in Table 2.

#### 3.2.1. Fluid Vector Change

**Figure 3:**
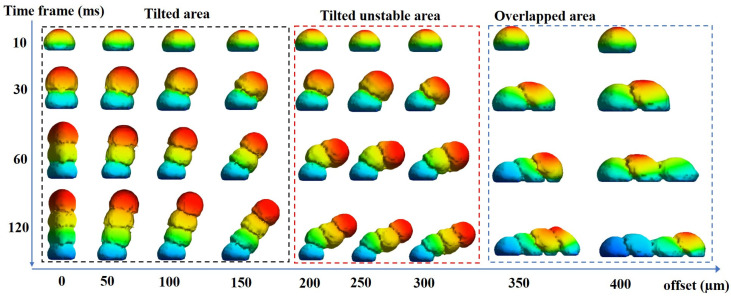
Morphology of microdroplets at different offsets.

The offset deposition of microdroplets was simulated at different offset amounts, as shown in Figure 3. With different microdroplet deposition spacing, it is obvious that the microdroplet formation is divided into three cases: when the offset is within the radius of the microdroplet, the tilt angle of the microdroplet formation is relatively stable and more controllable; after the offset is larger than the radius of the microdroplet, the first two microdroplets can still maintain a relatively good and stable state when the droplet is first formed, but the third microdroplet will have an unstable offset, which leads to the fourth microdroplet tilt being uncontrolled and at this time, if the deposition experiment is continued, a shape similar to an arch bridge will be formed; when the microdroplet spacing increases to close to the microdroplet diameter, the shape of the microdroplet will be completely flat on the substrate. There is a mesh module in the post-processing panel of the Flow-3D software, and when investigating the diameter of a sphere, the number of meshes occupied by the droplet is taken as the diameter when the closest approximation to a sphere is selected in the 3D mesh module.

From Figure 3, it is possible to observe the change in the morphology of the microdroplet fusion with time, and the second microdroplet is deposited on the first microdroplet at the right time during solidification to achieve better fusion. It is also possible to know that it is feasible to form a bridge-shaped channel, but tilting the channel from only one side will affect the formation of the channel due to the oscillation between the microdroplets during the formation and may lead to the collapse of the channel.

**Figure 4:**
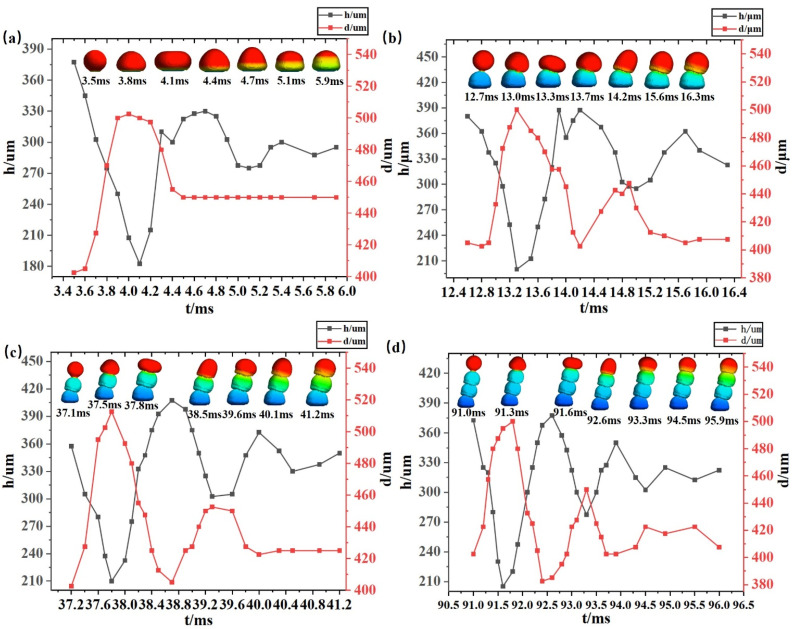
Variations in height and diameter of microdroplets during their fall at an interval of 100 μm. (**a**) represents the case of the first microdroplet changing after coming into contact with the substrate at 100 μm pitch; (**b**) represents the case of the second microdroplet changing after contact with the first microdroplet at 100 μm pitch; (**c**) represents the case of the third microdroplet changing after contact with the second microdroplet at 100 μm pitch; and (**d**) represents the case of the fourth microdroplet changing after contact with the third microdroplet at 100 μm pitch.

The simulation experiments selected from those presented in Figure 3 with an interval of 50 μm were further observed, and the variation of diameter versus height during the fall of the microdroplets was analyzed; the results are shown in Figure 4. The fusion both with the substrate and with the microdroplets produces an oscillation effect, thus changing the size of the microdroplets, but the overall fusion effect is positive. It is worth noting that the change in the droplet after 4.6 ms, shown in Figure 4a, is due to the fact that the lower part of the droplet is almost fixed and solidified after coming into contact with the substrate after 4.6 ms, and at this time, the temperature is already below the liquid phase line temperature and the lower part will not change, while the temperature of the upper part of the droplet is still changing and oscillating, so there will still be an oscillating change in the height of the droplet.

During the deposition of microdroplets on a substrate, a flat substrate is generally used. The microdroplet touches the substrate, spreads first, and then shrinks, and as heat is transferred to the substrate, the temperature of the microdroplet decreases and it solidifies. The metal in the spreading part solidifies rapidly, and the upper part of the microdroplet in the liquid state springs back and oscillates, and the solidification rate is greater than the time required for the oscillation process to form parallel ridges on the surface of the microdroplet. Microdroplets are deposited onto the solidified microdroplets, and the second microdroplet is preset to have a certain offset with respect to the previous one, and the fusion and spreading are more complicated than the collision, which can be divided into high and low frequencies. In the high-frequency case, the previous microdroplet is not completely solidified and the top part is in a liquid or liquid–solid state, which collides and fuses with the second microdroplet; in the low-frequency case, the previous microdroplet is completely solidified, but the temperature is higher than the substrate temperature, and fuses with the second microdroplet after collision by the heat carried by the microdroplet itself. Therefore, microdroplet fusion is better at high frequencies, but the morphology is more difficult to control. In this simulation, the low-frequency case is used, and the next microdroplet is deposited after the microdroplet solidifies.

**Figure 5:**
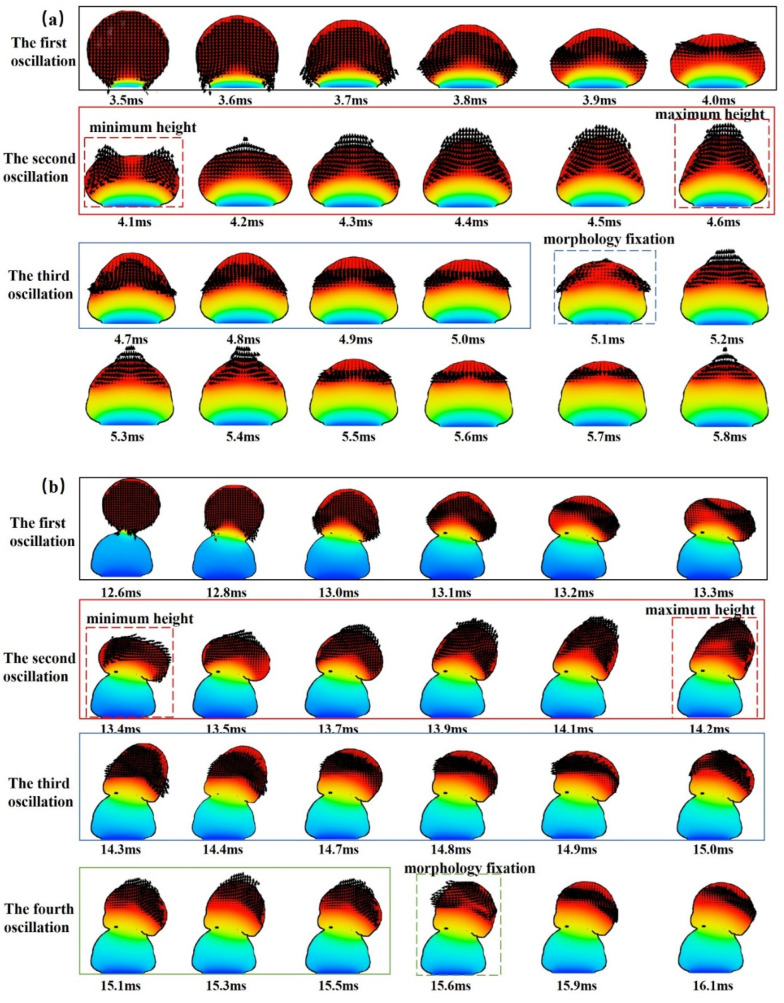
Plot of the fluid velocity vector variation in microdroplets. (**a**) Variation in velocity vector of microdroplet 1 in contact with the substrate. (**b**) Variation in velocity vector of microdroplet 2 in contact with microdroplet 1.

As shown in Figure 5a, the change in the fluid velocity vector after the contact of microdroplet 1 with the substrate is demonstrated. Microdroplet 1 touches the substrate at 3.5 ms, at which time the internal fluid vector arrow representing the velocity change (hereafter referred to as the vector arrow) changes direction only for the part that touches the substrate, and the vector arrow becomes smaller. After the bottom surface of microdroplet 1 comes into contact with the substrate, the high-temperature part starts to diffuse to the low-temperature region of the substrate, causing the bottom vector arrow to diffuse to both sides. The microdroplet gradually changes from a liquid to a solid state while the velocity decreases. During the first oscillation, the high-temperature part of microdroplet 1 gradually decreases and the vector arrows with the low-temperature intermixing part become sparse and smaller, indicating that the temperature velocity change of the high- and low-temperature intermixing part of the microdroplet is smaller and slower. On the contrary, the vector arrows of the outer part of the microdroplet gradually increase and become denser, indicating that the fluid velocity change in the outer part of the microdroplet is larger and faster. In the next 0.4 ms, microdroplet 1 transfers an increasing amount of temperature to the substrate, which causes the low-temperature region at the bottom of microdroplet 1 to start solidifying and setting; the width diffusion at the bottom of microdroplet 1 increases, and the bottom gradually solidifies. At this time, the vector arrows of both the outer part of the microdroplet and the high- and low-temperature intermingled parts gradually thin and decrease, but the overall vector arrow direction does not change until the end of the first oscillation. At the end of the first oscillation, microdroplet 1 spreads to its maximum width and starts the second oscillation. In the second oscillation starting from 4.1 ms, the morphology of microdroplet 1 starts to cluster towards the middle part. With time, the microdroplet becomes higher, and the vector arrows are sparse in the middle from the dense sides and are directed upward and converge toward the middle. At the same time, the interior changes slowly and the change near the outside of the microdroplet becomes faster. The second oscillation ends at 4.6 ms after microdroplet 1 reaches its maximum height. In the third oscillation starting from 4.7 ms, the morphological changes in the microdroplet are similar to the first oscillation, except that the changes are smaller, the vector arrows are fewer, and the oscillation speed is faster. The first oscillation took 0.6 ms, the second oscillation took 0.6 ms, and the third oscillation took 0.4 ms. After the completion of the third oscillation, the microdroplet had completed its transformation from solid–liquid to a solid state, and although the internal vector arrows were still changing, they no longer affected the formation of the microdroplet, but only continued the heat transfer.

Figure 5b shows the change in the fluid velocity vector after contact between microdroplet 2 and microdroplet 1. Because the heat dissipation after contact between microdroplet 2 and microdroplet 1 is longer than the time used when microdroplet 1 is in direct contact with the substrate, each oscillation time also becomes longer, and only a part of the change during the oscillation is captured in the figure, as follows:

Microdroplet 2 touches microdroplet 1 at 12.6 ms and starts spreading downward, and the internal vector arrows also start spreading outward to the contacting part of the two microdroplets. Unlike when microdroplet 1 touches the substrate, the vector arrows inside microdroplet 2 produce a unilateral aggregation phenomenon. The reason for this is that the vector arrows accumulate more on the right side due to the rightward shift of microdroplet 2 during the drop, which is consistent with the solidification pattern of microdroplet 2. At 13.1 ms, the bottom of microdroplet 2 starts to gradually solidify and the vector arrow stops going down; at 13.2 ms, the temperature spreads and the internal vector arrow changes, with the vector arrow of microdroplet 2 clustering to the upper right. This is because microdroplet 2 is shifted to the right during the descent, resulting in a faster temperature change on the right side than on the left side. At 13.3 ms, the liquid of microdroplet 2 partially diffuses to the preset offset position and completes the first oscillation. Unlike the first oscillation of microdroplet 1, the direction of the vector arrow of microdroplet 2 changes to the right and is not uniformly distributed during the oscillation. At 13.4 ms, microdroplet 2 starts to extend upward and the internal vector arrow also goes upward, while the second oscillation starts. The vector arrows are dense on the left side, indicating a faster rate of fluid change on the left side. At 13.7 ms, the vector arrows of microdroplet 2 gradually become uniform, the internal fluid velocity change tends to balance, and microdroplet 2 oscillates upward to the right. During the period from 13.9 ms to 14.2 ms, microdroplet 2 continues to extend to the maximum height to the upper right. Unlike the second oscillation of microdroplet 1, the fluid velocity variation at the beginning of the second oscillation of microdroplet 2 is not uniform, and the left side is faster than the right side. At 14.2 ms, the vector arrow in the lower right part of the interior rotates and the fluid velocity inside microdroplet 2 becomes slower on the left side and faster on the right side to complete the second oscillation. At 14.3 ms, microdroplet 2 starts the third oscillation and oscillates downward after the maximum height to achieve stability. Unlike the first oscillation of microdroplet 2, which extends downward, the first oscillation is more to the right and the second oscillation is more to the left. At this time, the vector arrow direction is more dense on the left side, and the rate of fluid change on the left side is greater than that on the right side, and the trend becomes more obvious with time. Unlike the third oscillation of microdroplet 1, microdroplet 2 is not fully set at the third oscillation, and the oscillation length is longer than that of microdroplet 1. The direction of the vector arrow inside also changes, from downward to leftward, and the distribution is not uniform. At 15.1ms, microdroplet 2 starts its fourth oscillation, and unlike the second oscillation, the upward extension is more to the left of microdroplet 2, and the offset of the vector arrow is smaller than the last one, but no rotation occurs. Unlike microdroplet 1, microdroplet 2 requires four oscillations to complete the fixation—one more than microdroplet 1. Microdroplet 2 takes 0.8 ms for the first oscillation, 0.9 ms for the second oscillation, 0.8 ms for the third oscillation, and 5 ms for the fourth oscillation, the total duration of which is greater than that of microdroplet 1. After completing the fourth oscillation, microdroplet 2 changes from a liquid to a solid state, and the vector arrow change continues, but it no longer affects microdroplet shaping, only heat transfer.

The change in the position of the microdroplet as it falls causes an effect in which the offset direction is the same as the direction of the position change; thus, the molding of the microchannel can be controlled. In addition, the oscillation situation after microdroplet 2 touches microdroplet 1 is slightly different from that when microdroplet 1 touches the substrate, because microdroplet 2 is in direct contact with microdroplet 1 and the situation is more complicated. However, by controlling the offset amount, the setting situation after the microdroplet goes down can be controlled. Whether the microdroplets are in contact with the substrate or the microdroplets are in contact with the microdroplets, the direction of change in the microdroplets during the oscillation is always along the direction of the normal vector of the microdroplets.

The law of fluid velocity change is similar to that of microdroplet formation. First, the fluid velocity gradually spreads to both sides, starting from the first contacted part. In the process of microdroplet shaping, the fluid velocity change on the outside of the microdroplet is always faster than in the center of the microdroplet, and both show uneven changes. Secondly, the changing velocity of the outer side shows a trend from large to small, and the direction of velocity change is consistent with the oscillation direction of the microdroplet, which changes downward and then upward repeatedly. When the microdroplet oscillates downward, the shape spreads out to the outer side; in the upward oscillation, the shape gathers and draws upward until the microdroplet is set. After that, the appearance of the microdroplet no longer changes, but the temperature inside is still changing over time. The rate of fluid change slows down once after each completed oscillation. It is noteworthy that each time an oscillation occurs and changes the direction of the vector arrows, the vector arrows inside the microdroplet change from an ordered arrangement to an uneven and unstable arrangement, and the direction changes as well. Since the solidification time of the microdroplet is usually shorter than the time required to complete all oscillations, the surface of the microdroplet shows oscillatory ripples after solidification.

#### 3.2.2. Temperature Gradient Change

**Figure 6:**
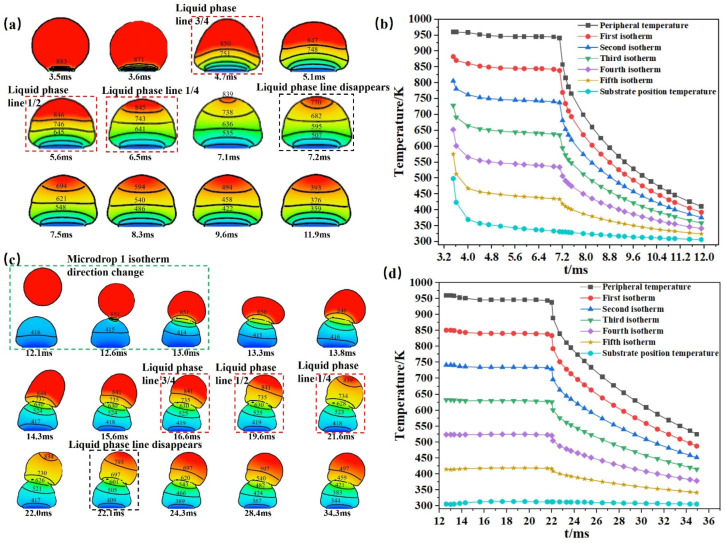
Temperature gradient variation graph. (**a**) Temperature gradient variation in microdroplet 1 in contact with the substrate. (**b**) Plot of temperature change at different moments in cooling of microdroplet 1. (**c**) Temperature gradient variation in microdroplet 2 in contact with microdroplet 1. (**d**) Plot of the temperature variation in microdroplet 2 at different moments of cooling.

Figure 6a shows the change in the internal temperature field after microdroplet 1 touches the substrate. After microdroplet 1 touches the substrate, the isotherm and temperature changes occur simultaneously, and the isotherm distribution is more dense at this time. This phenomenon is related to the rapid contact of the bottom with the substrate, resulting in a large temperature change in the bottom region, while the isotherms in the upper layer change at a slower rate. At 4.7 ms, the area of the temperature above the liquid phase line occupies 3/4 of the entire longitudinal cross section of the microdroplet, while at 5.6 ms, the area occupies 1/2 of the entire longitudinal cross section of the microdroplet. At this time, the two isotherms are clearly horizontal, and the temperature distribution in the upper area is relatively uniform. When 6.5 ms is reached, the area of the temperature above the liquid phase line occupies 1/4 of the whole microdroplet longitudinal section, and the isotherm spacing inside microdroplet 1 is almost the same, indicating that the temperature change rate inside microdroplet 1 is almost equal at this time. During the period from 3.5 ms to 7.1 ms, the isotherm scale changes slowly, but the morphology of the isotherm lines changes significantly, with each isotherm line varying in shape and direction, and the temperature field is relatively less stable during this time. By 7.2 ms, the liquid-phase line disappears and the temperature inside microdroplet 1 starts to change rapidly. After 7.5 ms, the isotherm line almost stops moving, the temperature field has stabilized, and the rate of temperature change inside changes from fast to slow. Using the time to drop 100 K as the unit of measurement, the times to drop from 694 K to 393 K are 0.6 ms, 1.3 ms, and 2.3 ms, respectively.

Figure 6c shows the temperature field change after microdroplet 2 is deposited on microdroplet 1, which has been fully fused to the substrate. Microdroplet 2 touches microdroplet 1 at 12.6 ms, when the isotherm changes, but the temperature has not yet changed. The temperature does not start to change until 13.3 ms. Unlike microdroplet 1, the isotherm inside microdroplet 2 spreads to both sides along the contact part of microdroplet 2 and microdroplet 1, respectively. In addition, the direction of change for the only remaining isotherm inside microdroplet 1 changes under the influence of microdroplet 2. Due to the heat transfer of microdroplet 2, the scale of the isotherm inside microdroplet 1 changes from decreasing to increasing. The liquid-phase line of the microdroplet only starts to move after microdroplet 2 is set, at 15.6 ms. At 16.6 ms, the liquid-phase line occupies 3/4 of the entire longitudinal cross section of the microdroplet; at 19.6 ms, it occupies 1/2 of the entire longitudinal cross section of the microdroplet. By 21.6 ms, the liquid-phase line occupies 1/4 of the entire longitudinal cross section of the microdroplet. However, the isothermal scale inside microdroplet 2 fluctuates very little until the liquid-phase line disappears. When the liquid phase line disappears at 22.1 ms, the temperature inside microdroplet 2 changes at a faster rate. At 24.3 ms, the isotherm line almost stops moving and the temperature field has stabilized. Using the time to drop 100 K as a unit of measurement, the drop from 697 K to 497 K took 4.1 ms and 5.9 ms, respectively.

The isotherm direction always follows the direction of the normal vector when the droplets are in contact with the substrate and between the microdroplets. However, when a microdroplet falls on an already-formed microdroplet and the drop pitch is changed so that the microdroplet forms at an angle, the isotherm direction of the formed microdroplet follows the normal vector direction of the next microdroplet. When the microdroplets are formed on the substrate, the isotherms tend to spread upward from the substrate, and initially, the isotherms are densely distributed in the part in contact with the substrate. When the microdroplets are in contact with each other, the isotherms tend to spread from the contacting part of the two microdroplets to the upper and lower sides at the same time, and the new microdroplets will affect the scale change of the isotherms inside the originally formed microdroplets, while the isotherms are densely distributed in the contacting part of the two microdroplets. Figure 6b,d, illustrate the change process of the internal isotherm scales of microdroplet 1 and microdroplet 2 during the cooling process. Thirty moments for each microdroplet were selected for comparison, and it can be observed that the greater the number of microdroplets, the longer the cooling time. The isotherm scale inside the microdroplet represents the temperature change of each part inside the microdroplet. When the liquid-phase line of the microdroplet has not disappeared, the internal temperature of microdroplet 1 in contact with the substrate changes slightly faster near the substrate than away from it; however, the internal temperature of microdroplet 2 in contact with microdroplet 1 changes very little, and all isotherms are approximately a straight line. When the liquid-phase line of the microdroplet disappears, the temperature change in the microdroplet is nonlinear, and the internal temperature of the microdroplet changes faster than before for a period of time, but the change in microdroplet 2 occurs more slowly than that in microdroplet 1 as a whole. When approaching the substrate temperature, the change in temperature slows down. The microdroplets and the substrate contact temperature began to fall, but at this time, because the temperature did not break through the liquid-phase line temperature, the rate of decline is relatively slow. With the passage of time, the temperature inside the microdroplet drops to the liquid-phase line temperature below the temperature of the microdroplet, the trend of the microdroplet temperature change significantly accelerates, the curvature of the curve becomes larger.

#### 3.2.3. Microchannel Top Change

The reason for the height imbalance between the two sides of the substrate is that the first side to complete the deposition of the next microdroplet will have an impact on the oscillation of the microdroplet. When the microdroplet spacing is large enough, it may have a small effect or even no effect on the microdroplet deposition on the first and second rows and on the oscillation of the basic disposition on the third row. On the fourth row, there may be small or no effects on microdroplet oscillation. For the third row of microdroplets, the height of the microdroplets decreased, and the width increased.

**Figure 7:**
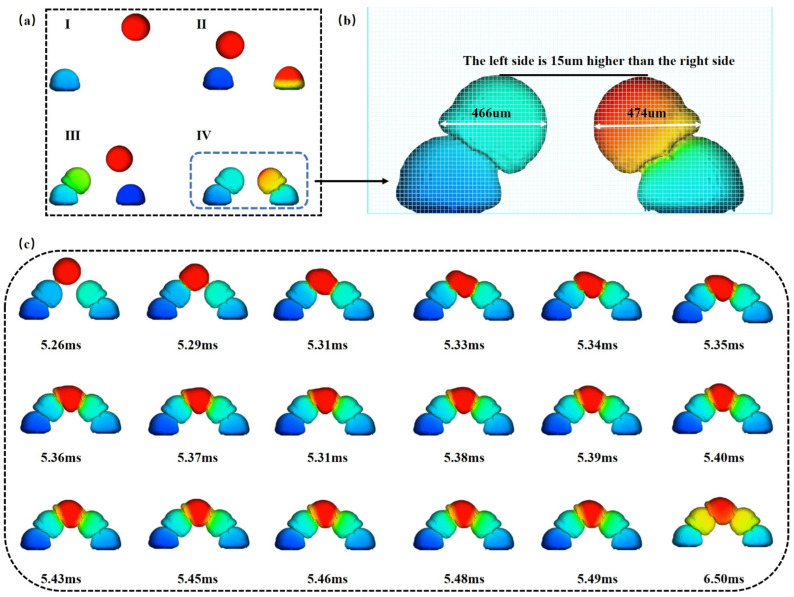
Changes in the last row of microdroplets as they fall in the microchannel molding simulation (**a**) Changes in the first four rows of microdroplets during microchannel deposition. I–IV are the sequence diagrams when depositing the first microdroplet to the fourth microdroplet at a time (**b**) Morphological differences between the third row of microdroplets and the fourth row of microdroplets. (**c**) Changes in the fifth row of microdroplets as they come into contact with the left and right substrates.

Because the microdroplet molding is divided into two sides alternately, the microdroplet molding pattern of the left and right sides is basically the same, which can be seen in Figure 7. When the last microdroplet is deposited on the microdroplet substrate that has been built in sequence, it can be observed that the microdroplet will first contact the left substrate, and then contact the right substrate, after which the microdroplet will expand the contact area with the substrate on both sides. At the same time, it will be oscillated and spread to the first downward oscillating process of the microdroplet. The left contact surface is always larger than the right side, and when the microdroplet extends downwards, the upper part of the microdroplet will also be oscillated from the left side to the right side; when the microdroplet extends downward to the minimum point, then it will start to oscillate upward and the upper part of the droplet will spread from the right side to the left side until the microdroplet reaches the upper part of the basic smooth equilibrium, and at that time, the droplet completes the finalized type.

## 4. Experimental Validation

**Figure 8:**
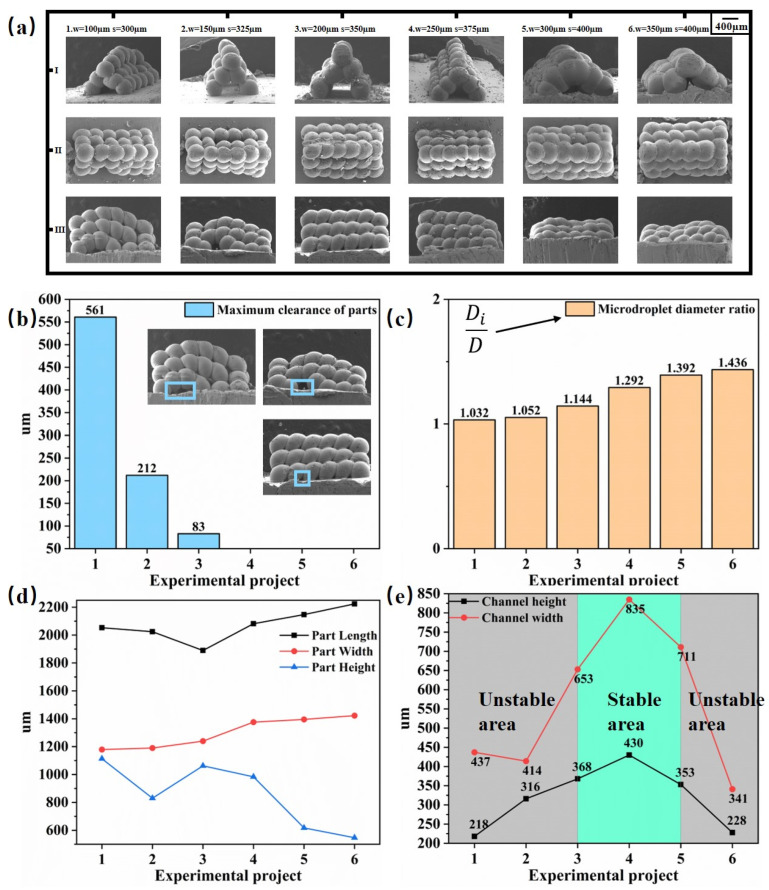
Metal microchannel formation experiments. (**a**) Schematic diagram of the microchannels formed by changing the deposition pitch (I: front view of the experiment formed at six pitches; II: top view of the experiment formed at six pitches; III: side view of the experiment formed at six pitches). (**b**) The maximum clearance of the six-spaced microchannel parts. (**c**) The ratio of the six-spaced microdroplet diameters, D_i_, to the standard microdroplet diameter, D (D = 400 µm). (**d**) External dimensions of the six-spaced microchannel parts. (**e**) The internal dimensions of the six-spaced microchannels.

Figure 8 shows the complete microchannel experimental procedure. In Figure 8a, six sets of microchannel parts molded at different pitches are shown: using the method in Figure 2b, microdroplet injection molding microchannel experiments were performed under this condition, and experimentally prepared microchannel parts were obtained. The channel structure can be observed in I, the morphology of the whole microchannel can be seen in II, and the sealing of the microchannel parts is well demonstrated in III. In Figure 8b, the maximum clearance of the side of the microchannel part is measured. In Figure 8c, the diameter, Di, of the first row of microdroplets at the top of the microchannel part is summarized by taking the average value compared to the diameter, D, of the microdroplets injected under the initial conditions of the experiment. In the first three sets of experiments, gaps were generated due to the unstable region of the spacing, s, as shown in Figure 3. Also, in the first set of experiments, the spacing, w, per layer and the spacing, s, per row of microdroplets were so small that the microdroplets could not be fully oscillated and spread. The diameter of the microdroplets was close to the experimental initial size, and the diameter ratio was close to 1, so gaps in the microchannel were present in each layer. When the spacing of each row, s, was increased to the spreading area shown in Figure 3, the droplets were fully oscillated and the gap disappeared, and the diameter ratio increased. In Figure 8d, the dimensions of all microchannel parts are measured, and it can be seen that the larger the microdroplet spread, i.e., the larger the diameter ratio of the microdroplets, the larger the length and width of the microchannel parts, but the smaller the relative height. In Figure 8e, the size of the internal channel of the microchannel parts is shown. In the last set of experiments involving the second row of microchannel parts, the layer spacing, w, is too large, leading to the second layer of microdroplets in contact with the substrate after oscillation, resulting in a significant reduction in the width of the microchannel. Obviously, the layer spacing, w, should not be too large, but when it is too small, the difference between the height and width of the microchannel is not large. This is because the second layer of microdroplets after oscillation spreading leads to the microchannel closing in advance, so more stable microchannel area parameters should be selected between the third and fifth group: the layer spacing, w, should be between 200 and 300 µm, and the row spacing, s, should be between 350 and 400 µm. The microchannels molded in this experiment were set as follows: the height of the part molded at w = 200 µm, s = 350 µm is 1062.4 µm, the width is 1240 µm, and the length is 1889.6 µm, and here, the height of the part is the largest, and the width and the length are the smallest; the height of the part molded at w = 300 µm, s = 400 µm is 617.6 µm and the width is 1395.2 um; at w = 300 µm, s = 400 µm, the molded height of the part is 617.6 µm, the width is 1395.2 µm, and the length is 2147.2 µm, and here, the height of the part is the smallest and the width and length are the largest.

## 5. Conclusions

A three-dimensional model for the continuous deposition of aluminum microdroplets on a substrate was developed using the VOF method. The simulation results of the model are consistent with the experimental results and provide a method to realize the continuous deposition of molten aluminum microdroplets on microchannels.

(1) Both the simulation and experimental results show that the fusion of microdroplets with the substrate and between microdroplets can be controlled by adjusting the parameters to meet the fusion degree during on-demand injection.
(2) Microdroplets are deposited onto the substrate, and then expand and then shrink. As heat is transferred to the substrate, the substrate temperature increases and the microdroplet temperature decreases and starts to solidify. Due to the rapid solidification of the unfolded part, the upper part of the microdroplet, which is still in the liquid state, will rebound and oscillate, and the solidification rate is greater than the oscillation rate of the microdroplet. As a result, the outer surface morphology of the microdroplet appears as an oscillating ripple caused by solidification.
(3) The deposition spacing has a more significant effect on the molded part. An insufficient microdroplet distance during deposition can result in excessive part buildup, while an excessive microdroplet distance can cause collapsed or unsealed parts.
(4) By simulating the observation of the oscillation phenomenon between microdroplets during microdroplet formation, the spacing can be used to control the degree of fusion between the microdroplets. This method is also applicable to the microdroplet injection of metals of other materials, which provides favorable conditions for manufacturing parts with different morphologies.

## Figures and Tables

**Table 1:** Thermo-physical properties of pure Al used in the simulation [39].

Physical Properties	Value
Density (kg/m^3^)	*ρ* = 2368
Dynamics viscosity (Pa·s)	*μ* = 1.257 × 10^−3^
Surface tension coefficient (N/m)	*σ* = 0.868
Heat conductivity coefficient (W/(m·K))	*k_s_* = 220 *k_l_* = 96.4
Specific heat capacity (J/(kg·K))	*C_ps_* = 1135 *C_pl_* = 1086
Latent heat for solidification (J/kg)	*L* = 397,500
Liquidus temperature (K)	*T_l_* = 934
Solidus temperature (K)	*T_s_* = 933
Static contact angle (°)	90

**Table 2:** Parameters related to numerical simulation experiments.

Parameters	Value
$\mathrm{Microdroplet}\;\mathrm{diameter}\;\left(\mathrm{mm}\right)$	0.6
$\mathrm{Microdroplet}\;\mathrm{drop}\;\mathrm{velocity}\;\left(m/s\right)$	0.35
$\mathrm{Microdroplet}\;\mathrm{drop}\;\mathrm{height}\;\left(\mathrm{mm}\right)$	3
$\mathrm{Microdroplet}\;\mathrm{initial}\;\mathrm{temperature}\;\left(K\right)$	960
$\mathrm{Substrate}\;\mathrm{temperature}\;\left(K\right)$	300
$\mathrm{Pulse}\;\mathrm{width}\;\left(\mathrm{ms}\right)$	0.4
$\mathrm{Environmental}\;\mathrm{oxidation}\;\left(\mathrm{PPM}\right)$	50~60

## Data Availability

The data that support the findings of this study are available from the corresponding author upon reasonable request.
